# The impact of obesity on severe disease and mortality in people with SARS‐CoV‐2: A systematic review and meta‐analysis

**DOI:** 10.1002/edm2.176

**Published:** 2020-08-14

**Authors:** Samuel Seidu, Clare Gillies, Francesco Zaccardi, Setor K. Kunutsor, Jamie Hartmann‐Boyce, Thomas Yates, Awadhesh Kumar Singh, Melanie J. Davies, Kamlesh Khunti

**Affiliations:** ^1^ Diabetes Research Centre Leicester General Hospital University of Leicester Leicester UK; ^2^ The NIHR Applied Research Collaboration (ARC) East Midlands Leicester General Hospital University of Leicester Leicester UK; ^3^ National Institute for Health Research Bristol Biomedical Research Centre University Hospitals Bristol NHS Foundation Trust University of Bristol Bristol UK; ^4^ Musculoskeletal Research Unit Translational Health Sciences Bristol Medical School Southmead Hospital University of Bristol Bristol UK; ^5^ Nuffield Department of Primary Care Health Sciences Centre for Evidence‐Based Medicine University of Oxford Oxford UK; ^6^ NIHR Leicester Biomedical Research Centre A Collaboration between University Hospitals of Leicester NHS Trust University of Leicester Leicester UK; ^7^ Diabetes & Endocrinology G.D Hospital & Diabetes Institute Kolkata India

**Keywords:** mortality, obesity, SARS‐CoV‐2, severe disease

## Abstract

**Background:**

Obesity accompanied by excess ectopic fat storage has been postulated as a risk factor for severe disease in people with SARS‐CoV‐2 through the stimulation of inflammation, functional immunologic deficit and a pro‐thrombotic disseminated intravascular coagulation with associated high rates of venous thromboembolism.

**Methods:**

Observational studies in COVID‐19 patients reporting data on raised body mass index at admission and associated clinical outcomes were identified from MEDLINE, Embase, Web of Science and the Cochrane Library up to 16 May 2020. Mean differences and relative risks (RR) with 95% confidence intervals (CIs) were aggregated using random effects models.

**Results:**

Eight retrospective cohort studies and one cohort prospective cohort study with data on of 4,920 patients with COVID‐19 were eligible. Comparing BMI ≥ 25 vs <25 kg/m^2^, the RRs (95% CIs) of severe illness and mortality were 2.35 (1.43‐3.86) and 3.52 (1.32‐9.42), respectively. In a pooled analysis of three studies, the RR (95% CI) of severe illness comparing BMI > 35 vs <25 kg/m^2^ was 7.04 (2.72‐18.20). High levels of statistical heterogeneity were partly explained by age; BMI ≥ 25 kg/m^2^ was associated with an increased risk of severe illness in older age groups (≥60 years), whereas the association was weaker in younger age groups (<60 years).

**Conclusions:**

Excess adiposity is a risk factor for severe disease and mortality in people with SARS‐CoV‐2 infection. This was particularly pronounced in people 60 and older. The increased risk of worse outcomes from SARS‐CoV‐2 infection in people with excess adiposity should be taken into account when considering individual and population risks and when deciding on which groups to target for public health messaging on prevention and detection measures.

**Systematic review registration:** PROSPERO 2020: CRD42020179783.

## 1 BACKGROUND

The severe acute respiratory syndrome coronavirus 2 (SARS‐CoV‐2), initially reported in November 2019 in Wuhan, China, has now claimed over 300 000 lives globally^1^ and devastated the global economy. It continues to severely affect the social fabrics of most counties. The unprecedented projected mortality and economic devastation caused by the virus has led to global research efforts to identify people at greatest risk of developing critical illness and dying. Chen et al identified older age, male gender and comorbidities such as hypertension, diabetes, cardiovascular disease and chronic lung disease as risk factors for severe disease when they compared the characteristics of 113 (14.4%) patients who had died of the disease with those of 161 patients who recovered.^2^ Recent studies have increasingly described obesity as an associating factor for people at an increased risk of severe disease.^3^, ^4^, ^5^, ^6^ The risk of many chronic diseases such as cardiovascular disease, cancer, diabetes and the mortality in individuals with these diseases increases significantly in people with obesity or overweight.^7^ With the World Health Organization estimating that more than 1.9 billion adults worldwide have overweight or obesity,^8^ any causal relationship or association between obesity and severe disease from SARS‐CoV‐2 has the potential to claim even more lives globally.

There may be pathophysiological mechanisms for worse outcomes in people with excess adipose tissues.^9^ Excess adipose tissue leads to local insulin resistance and may stimulate inflammation, functional immunologic deficit and a pro‐thrombotic disseminated intravascular coagulation with associated high rates of venous thromboembolism.^10^, ^11^

Conversely, the phenomenon of the ‘obesity paradox’ has emerged from epidemiological data in recent years to suggest counterintuitively that people with overweight and obesity may have a better prognosis than those with BMI values in the normal range. This phenomenon has thus far been described in patients with hypertension, heart failure, coronary artery disease, peripheral artery disease and other cardiovascular and noncardiovascular conditions.^12^, ^13^ The phenomenon seems more convincing in older populations where weight and muscle mass start to decline at advanced age. Sarcopenia is a major contributor to age‐related frailty.^14^ It is therefore possible that people with overweight and obesity, especially in older populations, could have less severe disease and mortality compared to individuals with normal weight.

In this context, whether obesity has better or worse impact on the occurrence of severe disease or death in people with SARS‐CoV‐2 is uncertain. A recent systematic review on this topic identified only three studies and concluded that obesity is an independent risk and prognostic factor for SARS‐CoV‐2. The review was limited by the small number of included studies, the absence of some key data and the lack of quantitative synthesis.^15^ In order to attempt to quantify the relationship between raised body weight and severe outcomes from COVID‐19, we conducted a systematic review and meta‐analysis to determine whether people with overweight or obesity and with SARS‐CoV‐2 have different outcomes compared to those within normal weight thresholds.

## 2 METHODS

### 2.1 Data sources and search strategy

This review was conducted and reported based on PRISMA and MOOSE guidelines^16^, ^17^ (Appendices S1 and S2) and in accordance with a protocol which has been registered with PROSPERO, (CRD42020179783). We searched MEDLINE, Embase, Web of Science and The Cochrane Library from inception to 16 May 2020 for studies reporting on relationships between overweight and obesity and the clinical outcomes in patients with COVID‐19. The computer‐based searches combined free and MeSH search terms and key words related to the exposures (such as ‘Body Mass Index’, ‘BMI’ ‘Body Weight’ ‘Obesity’.) and population (eg ‘COVID‐19’, ‘SARS‐CoV‐2’) in humans. The search strategy was limited to English language given the potential for duplicate reporting of same study participants.^18^ The full search strategy is reported in Appendix S3. The titles and abstracts of retrieved citations were initially screened by one reviewer (SS) for potential eligible studies based on the inclusion criteria. Full texts of potential eligible abstracts were then acquired for the detailed evaluation by 2 reviewers (SS and SKK). To potentially identify articles missed by the search strategy, reference lists of relevant articles were manually scanned.

### 2.2 Study selection and eligibility criteria

The protocol was designed to include all observational studies (prospective cohort, retrospective, nested case‐control and case‐control), clinical studies, nonrandomized controlled trials (RCTs) and RCTs reporting a relationship between obesity and the clinical outcomes in patients with COVID‐19. Article types such as opinion pieces, consensus reports, single case studies and narrative reviews were excluded. Outcomes evaluated included severe disease (defined as intensive care unit (ICU) care, SpO_2_ < 90%, requiring supplemental O_2_) and mortality. No limits were placed on the study follow‐up duration.

### 2.3 Data extraction and quality assessment

Using a predesigned data extraction form which had been piloted by one reviewer (SKK), data on patient characteristics (eg average age, sex, percentage of males, location); study design; BMI (means, medians, standard deviations [SDs] and interquartile ranges [IQRs] in each outcome group); outcomes; and risk estimates where available (relative risks [RR] with 95% confidence intervals [CIs], hazard ratios [HRs] and odds ratios [ORs]) were extracted. The nine‐star Newcastle‐Ottawa Scale (NOS),^19^ a validated tool for assessing the quality of nonrandomized studies, was used to assess the methodological quality of the studies. This tool measures the quality of evidence from a score of zero to nine (higher scores = better), based on three predefined domains including the following: (a) selection of participants; (b) comparability; and (c) ascertainment of outcomes of interest.

### 2.4 Statistical analysis

Mean differences (95% CIs) for comparing mean levels of body mass index (BMI) across outcomes and RRs (95% CIs) for associations between BMI categories and outcomes were used as summary measures across studies. The reported HRs were assumed to approximate the same measure of RR. Multivariable‐adjusted risk estimates were used for pooling if available, otherwise crude RRs were used as reported. For data reported as medians, standard errors, ranges and 95% confidence intervals (CIs), means and standard deviations were estimated using methods as described by Hozo and colleagues.^20^ Due to the different cut‐offs used for BMI by the included studies, we employed the risk comparison: ≥25 vs <25 kg/m^2^, to ensure consistency in the pooling approach and enhance comparability and interpretation of findings. Subsidiary analyses employed the cut‐off > 35 vs <25 kg/m^2^. Similarly, as there was heterogeneous reporting of outcomes, we categorized them into two main groups for consistency: (a) severe illness (requiring intensive care unit (ICU) care, SpO_2_ < 90%, and requiring supplemental O_2_) and (b) mortality. Random effects models using the inverse variance weighted method (DerSimonian and Laird) were used to combine RRs to minimize the effect of heterogeneity. Heterogeneity was assessed and quantified using the Cochrane *χ* ^2^ statistic and the *I* ^2^ statistic.^21^ Prespecified study‐level characteristics such as geographical location, average age at baseline and study quality were explored as sources of heterogeneity, using stratified analysis and random effects meta‐regression.^22^ We assessed for evidence of publication bias using visual inspection of Begg's funnel plots and Egger's regression symmetry tests. STATA release MP 16 (StataCorp LP) was used for all statistical analyses.

## 3 RESULTS

### 3.1 Study identification and selection

**FIGURE 1:**
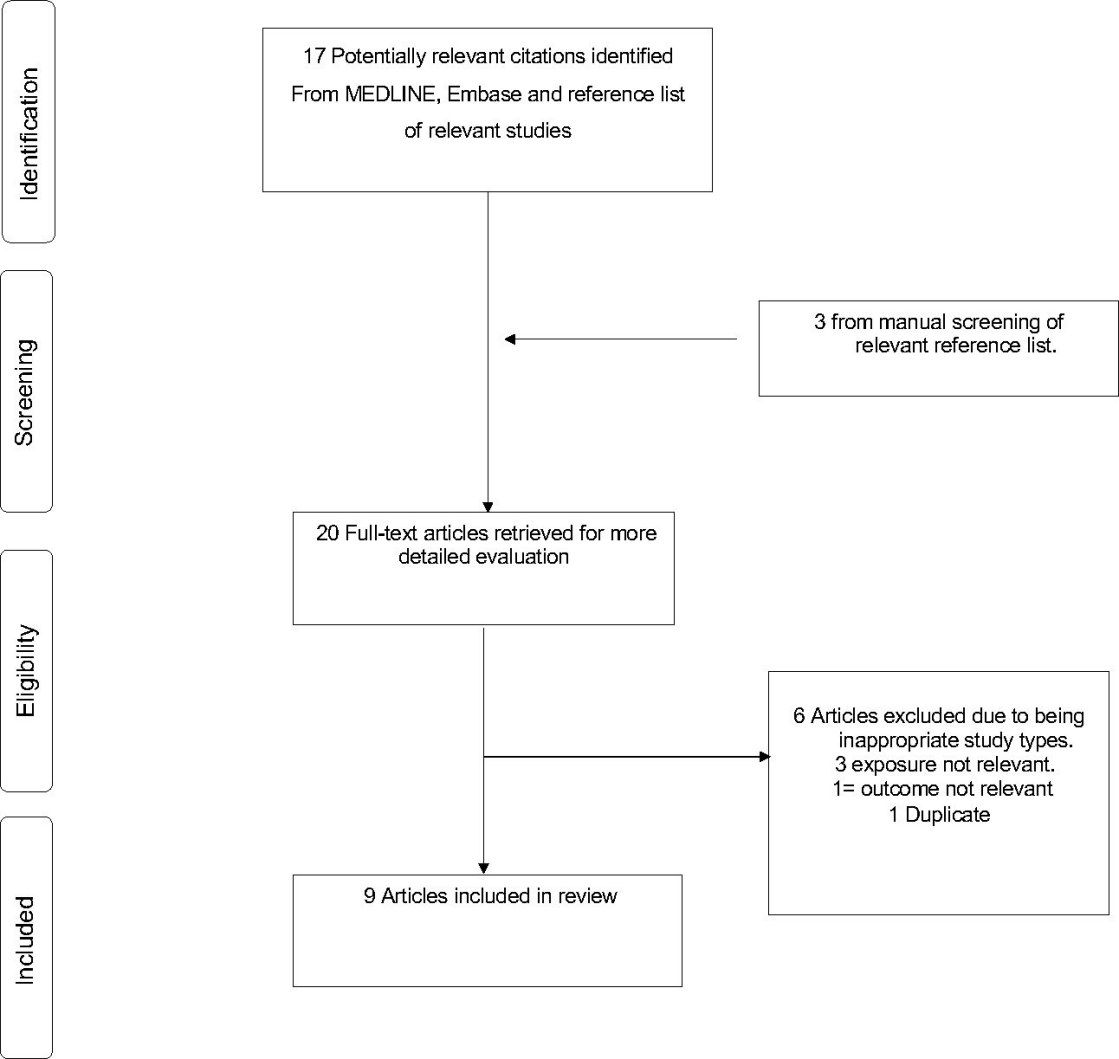
Selection of studies included in the meta‐analysis

The flow of studies through the review process is presented in Figure 1. A total of 17 articles were identified from the search of databases and another three from manual scanning of reference lists of relevant studies. After initial screening based on titles and abstracts, nine articles remained for full‐text evaluation. Eleven articles were excluded because (a) the exposure was not relevant (n = 3), (b) outcome was not relevant (n = 1), (c) duplicate (n = 1 and (d) inappropriate article types such as opinion pieces, consensus reports single case studies and narrative reviews (n = 6).

### 3.2 Study characteristics and quality

All nine studies comprised of observational cohorts (8 retrospective and 1 prospective) and comprised of 4920 patients with COVID‐19, of which 841 developed severe illness and 136 died (Table 1). Four studies were based in Asia (China and Singapore) (n = 549), three from Europe (France and Italy) (n = 653) and two from North America (USA) (n = 3718). The average age at baseline ranged from approximately 43 to 64 years, with a weighted mean (SD) of 56.8 (8.3) years. All studies enrolled both male and female patients. The overall NOS methodological quality scores of studies ranged from 4 to 7.

**TABLE 1:** Baseline characteristics of included studies of COVID‐19 patients

Author, year of publication	Source of participants	Country	Date of data collection	Mean/median Age (y)	Male %	Total participants	No. of outcomes	Reported outcomes	Derived outcome	NOS score
Zheng, 2020	Three hospitals in Wenzhou	China	17 January‐11 February 2020	47	25.8	66	19	In‐hospital mortality	Mortality	7
Kalligeros 2020	Hospital Network in Rhode Island	USA	17 February‐5 April 2020	60	63	103	41	ICU care	Severe illness	7
Kalligeros 2020	Hospital Network in Rhode Island	USA	17 February‐5 April 2020	60	63	103	29	Invasive mechanical Ventilation	Severe illness	7
Simonnet 2020	Roger Salengro Hospital, Lille	France	27 February‐5 April 2020	60	73	124	18 (30 for severe obesity)	invasive mechanical ventilation	Severe illness	7
Peng 2020	Western district of Union Hospital in Wuhan	China	20 January‐15 February	62	47.32	112	17	Mortality	Mortality	5
Ong 2020	National Centre for Infectious Diseases, Singapore	Singapore	NR	54.9	56	91	4	Mortality	Mortality	5
Ong 2020	National Centre for Infectious Diseases, Singapore	Singapore	NR	54.9	56	91	43	ICU care	Severe illness	5
Giacomelli, 2020	Luigi Sacco Hospital	Italy	21 February‐19 March 2020	61	69.1	233	48	Mortality		6
Lighter, 2020	Academic Hospital in New York	USA	4 March‐4 April 2020	59		3615	431	ICU care	Severe illness	4
Rottoli, 2020	8 tertiary and district hospitals	Italy	28 February‐28 March 2020	64.2	65.2	296	110	Respiratory failure	Severe illness	6
Rottoli, 2020	8 tertiary and district hospitals	Italy	28 February‐28 March 2020	64.2	65.2	296	67	Mortality	Mortality	6
Wu, 2020	First People's Hospital of Yancheng City, the Second People's Hospital of Fuyang City, the Second People's Hospital of Yancheng City and the Fifth People's Hospital of Wuxi	China	20 January‐19 February 2020	43.1	53.9	280	83	Severe disease	Severe illness	6

Abbreviations: ICU, intensive care unit; NR, not reported.

### 3.3 BMI and risk for severe illness and mortality

**FIGURE 2:**
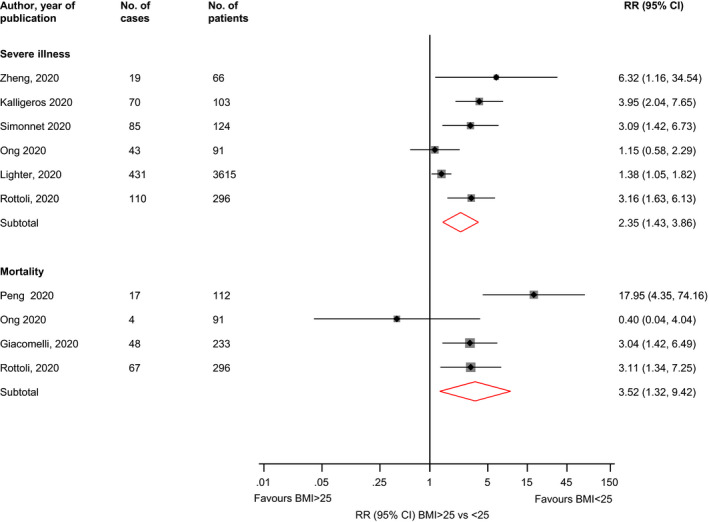
Associations of BMI ≥ 25 vs <25 kg/m^2^ with risk of severe illness and mortality in COVID‐19 patients. BMI, body mass index; CI, confidence interval (bars); RR, relative risk

**FIGURE 3:**
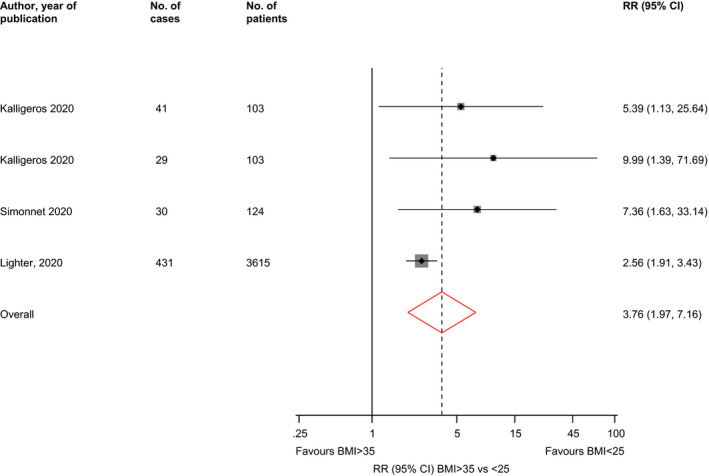
Associations of BMI > 35 vs <25 kg/m^2^ with risk of severe illness in COVID‐19 patients. BMI, body mass index; CI, confidence interval (bars); RR, relative risk

Comparing BMI ≥ 25 vs <25 kg/m^2^, the RRs (95% CIs) of severe illness (n = 6 studies) and mortality (n = 4 studies) were 2.35 (1.43‐3.86; *I* ^2^ = 71%; 95% CI 33, 88%; *P* for heterogeneity = .004) and 3.52 (1.32‐9.42; *I* ^2^ = 66%; 95% CI 0, 88%; *P* for heterogeneity = .03), respectively (Figure 2). In pooled analysis of three studies, the RR (95% CI) of severe illness comparing BMI > 35 vs <25 kg/m^2^ was 7.04 (2.72‐18.20; *I* ^2^ = 29%; 95% CI 0, 74%; *P* for heterogeneity = 0.24) (Figure 3).

**FIGURE 4:**
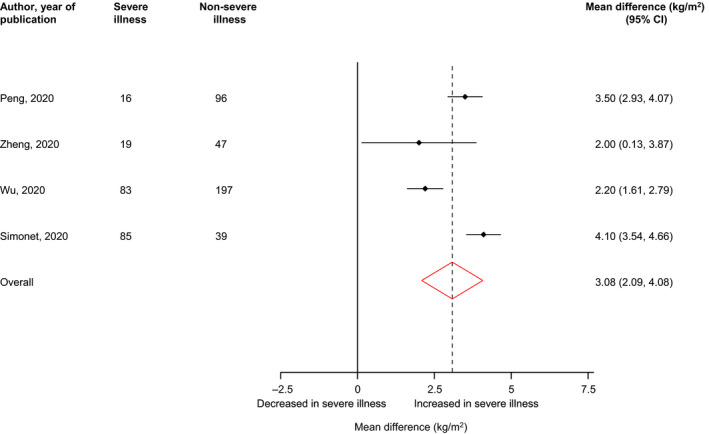
BMI levels in COVID‐19 patients with or without severe illness. BMI, body mass index; CI, confidence interval (bars); RR, relative risk

Figure 4 shows BMI levels in patients with severe illness compared with their controls (those without severe illness). Pooled analysis of four studies each showed significantly higher BMI levels in COVID‐19 patients with severe illness compared to patients without severe illness: mean difference (95% CI) of 3.08 kg/m^2^ (2.09, 4.08; *P* < .01; *I* ^2^ = 87%; 95% CI 69, 95%; *P* for heterogeneity < .01).

### 3.4 Subgroup analyses and observed heterogeneity

**FIGURE 5:**
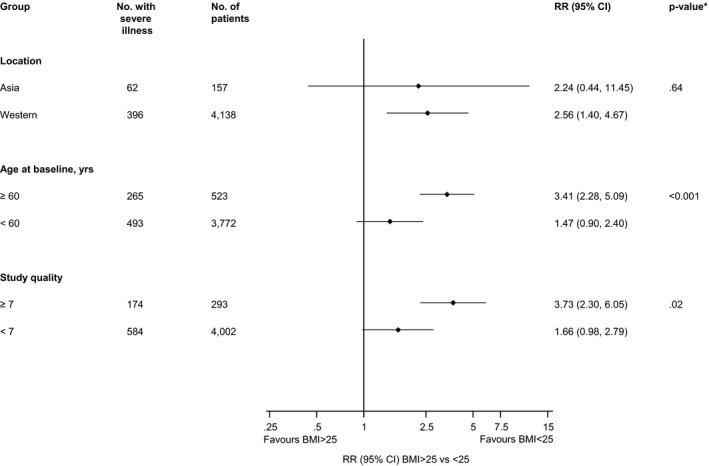
Associations of BMI ≥ 25 vs <25 kg/m^2^ with risk of severe illness in COVID‐19 patients, by study‐level characteristics. BMI, body mass index; CI, confidence interval (bars); RR, relative risk

The substantial heterogeneity between the contributing studies reporting on the association between BMI ≥ 25 vs <25 kg/m^2^ and risk of severe illness may be partly explained by age (*P*‐value for meta‐regression < .001) and study quality (*P*‐value for meta‐regression = .02) (Figure 5). In subgroup analyses, BMI ≥ 25 kg/m^2^ was associated with a statistically significant increased risk of severe illness in older age groups (≥60 years); the point estimate was lower and the difference was no longer statistically significant in younger age groups (<60 years). In a second subgroup analysis, BMI ≥ 25 kg/m^2^ was associated with statistically significant increased risk of severe illness in higher quality studies (NOS ≥ 7), whereas the point estimate was lower and no longer statistically significant in studies with a lower quality score (NOS < 7) (Figure 5). Given the reduced heterogeneity in the comparison of BMI > 35 kg/m^2^ vs <25 kg/m^2^ and the markedly higher point estimate in this comparison, it is also likely that difference in BMI ranges and thresholds between studies contributed to the high levels of statistical heterogeneity in both the analyses comparing BMI ≥ 25 vs <25 kg/m^2^.

The reasons for the observed heterogeneity in our analysis comparing BMI levels in people with severe illness compared to those without is unclear; due to the small number of studies, subgroup analyses were not appropriate. However, despite high levels of statistical heterogeneity, all studies included in this analysis found statistically significant increases in BMI in people with severe disease; the direction of effect was consistent and differences in the magnitude of effect drove the statistical heterogeneity in this case.

## 4 DISCUSSION

Using a systematic review and meta‐analytical approach, we have summarized the available studies of SARS‐CoV‐2 patients that have assessed relationships between raised BMI and the adverse clinical outcomes of severe illness and mortality. Our main finding of people with increased BMI with SARS‐CoV‐2 being at increased risk of severe disease and death reinforces the findings from a smaller review^15^ indicating that obesity is an important risk factor for severe SARS‐CoV‐2 disease. In addition, several other opinion pieces, editorials and reviews^9^, ^23^, ^24^, ^25^ have recently suggested that after adjusting for comorbidities, obesity is a significant factor associated with in‐hospital death in SARS‐CoV‐2. Severe obesity has now been recognized by the Centers for Disease Control and Prevention (CDC) as a common clinical risk factor for worse outcomes and higher mortality in people with SARS‐CoV‐2.^26^ These findings in relation to SARS‐Cov‐2 and excess adiposity are consistent with associations seen in other infectious diseases, including the H1N1 pandemic.^27^ People with overweight or obesity are generally more likely to have more severe infections, decreased responses to treatments and an increased risk of death.^27^

We have shown that people with higher BMI have an increased risk of severe disease in older age groups (≥60 years); the association was less clear in younger people. In a recent analysis of 265 patients with COVID‐19, Kass et al^28^ described a significant inverse correlation between age and BMI, in which younger individuals admitted to hospital were more likely to be obese. The authors suggest that, on this basis, public messaging to younger adults be clear that even at younger ages, they are still vulnerable to COVID‐19. Though our data did not find the same pattern as Kass et al, we do not disagree with this messaging. The average age at baseline in our population ranged from approximately 43 to 64 years, which is relatively young. Despite this relatively young population, 17% of our population had severe disease or death.

### 4.1 Possible mechanisms explaining the link between raised body weight and worse outcomes from COVID‐19

Several mechanisms could explain why raised body weight predisposes patients with SARS‐CoV‐2 to severe disease. First, raised body weight increases mechanical pressure on chest and abdomen causing diaphragmatic embarrassment, thus restricting pulmonary function, especially when lying supine. This causes a decrease expiratory reserve volume, functional capacity and respiratory system compliance.^24^

Second, obesity is associated with type 2 diabetes and hypertension, both of which constitute important risk factors for cardiovascular disease and are very prevalent in SARS‐CoV‐2 patients.^29^ Similarly, obesity is one of the leading risk factors for atrial fibrillation and this is a common condition present in severe forms SARS‐CoV‐2.^30^ Given limitations in our data, we were unable to ascertain the extent to which pre‐existing conditions contribute to the observed association between raised BMI and worse outcomes from COVID‐19.

Thirdly, obese patients have excess ectopic visceral fat, which correlates with a cluster of metabolic abnormalities.^31^ Visceral fat represents a metabolically active organ and has been strongly related to insulin sensitivity^32^, ^33^ and CVD. Visceral fat adipocytes are insulin‐resistant cells within a network of blood capillaries and infiltrating inflammatory cells.^34^ Inflammatory cells within the visceral fat may play a role in adipocyte behaviour as a source of hormones and cytokines, called adipokines, with proinflammatory and proatherogenic action. Obesity is therefore proinflammatory. Thus, in a patient with an already underlying proinflammatory state, the exposure to a viral infection in which early reports suggest that a cytokine storm syndrome^35^ occurs, associated with an overproduction of immune cells and proinflammatory cytokines (eg IL‐6, IL‐10 and TNF‐α),^36^ is likely to predispose to more severe disease.

Fourthly, SARS‐CoV‐2 binds with angiotensin‐converting enzyme 2 (ACE2) receptors on the cell surfaces. Excess visceral fat is linked to insulin resistance and a heightened activity of aldosterone system (RAAS), which is linked with worse outcomes in COVID‐19.^37^ Even though the lung is the main entry point for COVID‐19, it has been noted that there is an increased ACE2 expression in adipose tissue, thus making it a more vulnerable target for COVID‐19 infection.^38^ The presence of ACE2 may enable the entry of SARS‐CoV‐2 into adipocytes, which makes adipose tissue an important viral reservoir for greater viral shedding suggesting potential for great viral exposure,^38^ through spread to other organs.^39^

Finally, obesity enhances thrombosis,^40^, ^41^ and this increases with the severity of obesity.^42^ The mechanistic pathway implicated in obesity‐related hypercoagulability includes the actions of adipocytokines, coagulation factors hyperactivity and increased inflammation (tumour necrosis factor [TNF], interleukin‐6 [IL‐6]) among others.^43^ COVID‐19 has been noted to be associated with pro‐thrombotic disseminated intravascular coagulation and higher rates of venous thromboembolism, leading to worse outcomes.^38^ Therefore, increased hypercoagulability and thrombosis in COVID‐19 patients may contribute to additive effects of obesity and SARS‐CoV‐2 infection.

### 4.2 Strengths and limitations

Our analysis has several strengths. Our comprehensive search strategy yielded the highest number of published articles on this topic to date and evaluated the relationship of overweight and obesity on adverse outcomes in people with COVID‐19. We were able to transform reported risk estimates from majority of contributing studies to a consistent level to allow combination of estimates across studies, which enhanced interpretation of the overall findings. Our subgroup analysis revealed that older age and higher quality studies explained some of the heterogeneity observed; different BMI thresholds and ranges across the included studies also likely contributed to the observed heterogeneity. We included studies with populations from a wide range of regions, unlike other reviews on COVID 19, which tend to have patients over‐represented from Asia.

Our study has some limitations, most of which relate to the quality and reporting of the included studies as well as to differences between studies which contributed to statistical heterogeneity. The definitions of severe illness varied across the studies; hence, the use of a composite measure was applied. BMI ranges also varied across studies. Ethnicity, comorbid conditions and socio‐economic status are linked to obesity and to worse COVID‐19 outcomes, but we were unable to investigate these links due to insufficient data. For the same reason, the role of comorbidities has not been accounted for in this analysis. All of these factors could be important drivers of observed links between raised BMI and worse outcomes with COVID‐19. In addition, sample sizes were limited; hence, there was inadequate power to reliably evaluate the nature and magnitude of the associations. Finally, there is a potential for some overlapping to have occurred in the data, though this is unlikely as the cases were all from different centres. For these reasons, though the data are consistent on the increased risk of severe disease from COVID‐19 in people with raised body weight, we are unable to explore causality or to precisely quantify risks. Research into COVID‐19 is in its relative infancy, and as more data emerge, it is important these are transparently reported and take into account a range of factors related to COVID‐19 outcomes. In the meantime, the policy implications of the observed link between raised BMI and COVID‐19 need careful consideration.

## 5 CONCLUSION

Despite important differences between studies and relatively small sample sizes, data consistently suggest raised body weight is a risk factor for severe disease and death with COVID‐19. This is particularly pronounced in older age groups and in higher BMI ranges. The increased risk of worse outcomes from SARS‐CoV‐2 infection in people with overweight and obesity should be taken into account when considering individual and population risks and when deciding on which groups to target with public health messaging and increased prevention and detection measures.

## CONFLICTS OF INTEREST

KK reports personal fees from Berlin‐Chemie AG/ Menarini Group, personal fees from Sanofi‐Aventis, personal fees from Servier, personal fees from Boehringer Ingelheim, grants from Pfizer, grants from Boehringer Ingelheim, grants from AstraZeneca, grants from Novartis, grants from Novo Nordisk, grants from Sanofi‐Aventis, grants from Lilly, grants from Merck Sharp & Dohme, grants from Servier, outside the submitted work. SS reports personal fees from Amgen, personal fees from Astrazeneca, personal fees from NAPP, personal fees from Lilly, personal fees from Merck Sharp & Dohme, personal fees from Novartis, personal fees from Novo Nordisk, personal fees from Roche, personal fees from Sanofi‐Aventis, personal fees from Boehringer Ingelheim, grants from AstraZeneca, grants from Sanofi‐Aventis, grants from Servier, grants from Janssen, outside the submitted work. MJD reports personal fees and grants from Boehringer Ingelheim, Janssen, Novo Nordisk and Sanofi, and personal fees from AstraZeneca, Eli Lilly, Gilead Sciences Ltd., Intarcia/Servier, Merck Sharp & Dohme, Mitsubishi Tanabe Pharma Corporation and Takeda Pharmaceuticals International Inc.

## AUTHOR CONTRIBUTION

Samuel Seidu: study design, data collection, data analysis, data interpretation, figures and writing. Clare Gillies: data collection, figures, data interpretation and writing. Francesco Zaccardi: figures, data interpretation and writing. Setor K. Kunutsor: figures, data analysis, data interpretation and writing. Jamie Hartmann‐Boyce: data interpretation and writing. Thomas Yates, Awadhesh Kumar Singh and Melanie J. Davies: data interpretation and writing. Kamlesh Khunti: study design, data interpretation, figures and writing.

## Supporting information

Supinfo

## Data Availability

The corresponding author had full access to all the data in the studies used for the analysis and takes responsibility for the accuracy of the data analysis. The studies are all publicly available in the publications. The data that support the findings of this study as listed in the reference list, are openly available on PUBMED.
