# Supplementary material for: The impact of obesity on severe disease and mortality in people with SARS‐CoV‐2: A systematic review and meta‐analysis
Source: Endocrinol Diabetes Metab. 2020 Aug 14;4(1):e00176. doi: 10.1002/edm2.176 (PMC7460942; doi:10.1002/edm2.176)
Supplement: Supplementary file 1 — Supinfo [file EDM2-4-e00176-s001.docx]

**SUPPLEMENTARY MATERIAL**

| **Appendix 1** | PRISMA checklist |
| --- | --- |
| **Appendix 2** | MOOSE checklist |
| **Appendix 3** | MEDLINE literature search strategy |
| **Appendix 4** | Associations of obesity with mortality in COVID-19 |

**Appendix 1:** PRISMA checklist

| **Section/topic** | **Item No** | **Checklist item** | **Reported on page No** |
| --- | --- | --- | --- |
| **Title** | | | |
| Title | 1 | Identify the report as a systematic review, meta-analysis, or both | 1 |
| **Abstract** | | | |
| Structured summary | 2 | Provide a structured summary including, as applicable, background, objectives, data sources, study eligibility criteria, participants, interventions, study appraisal and synthesis methods, results, limitations, conclusions and implications of key findings, systematic review registration number | 2 |
| **Introduction** | | | |
| Rationale | 3 | Describe the rationale for the review in the context of what is already known | Introduction |
| Objectives | 4 | Provide an explicit statement of questions being addressed with reference to participants, interventions, comparisons, outcomes, and study design (PICOS) | Introduction |
| **Methods** | | | |
| Protocol and registration | 5 | Indicate if a review protocol exists, if and where it can be accessed (such as web address), and, if available, provide registration information including registration number | Methods |
| Eligibility criteria | 6 | Specify study characteristics (such as PICOS, length of follow-up) and report characteristics (such as years considered, language, publication status) used as criteria for eligibility, giving rationale | Methods |
| Information sources | 7 | Describe all information sources (such as databases with dates of coverage, contact with study authors to identify additional studies) in the search and date last searched | Methods |
| Search | 8 | Present full electronic search strategy for at least one database, including any limits used, such that it could be repeated | Appendix 3 |
| Study selection | 9 | State the process for selecting studies (that is, screening, eligibility, included in systematic review, and, if applicable, included in the meta-analysis) | Methods |
| Data collection process | 10 | Describe method of data extraction from reports (such as piloted forms, independently, in duplicate) and any processes for obtaining and confirming data from investigators | Methods |
| Data items | 11 | List and define all variables for which data were sought (such as PICOS, funding sources) and any assumptions and simplifications made | Methods |
| Risk of bias in individual studies | 12 | Describe methods used for assessing risk of bias of individual studies (including specification of whether this was done at the study or outcome level), and how this information is to be used in any data synthesis | Methods |
| Summary measures | 13 | State the principal summary measures (such as risk ratio, difference in means). | Methods |
| Synthesis of results | 14 | Describe the methods of handling data and combining results of studies, if done, including measures of consistency (such as I^2^ statistic) for each meta-analysis | Methods |
| Risk of bias across studies | 15 | Specify any assessment of risk of bias that may affect the cumulative evidence (such as publication bias, selective reporting within studies) | Methods |
| Additional analyses | 16 | Describe methods of additional analyses (such as sensitivity or subgroup analyses, meta-regression), if done, indicating which were pre-specified | Methods |
| **Results** | | | |
| Study selection | 17 | Give numbers of studies screened, assessed for eligibility, and included in the review, with reasons for exclusions at each stage, ideally with a flow diagram | Results, Figure 1 |
| Study characteristics | 18 | For each study, present characteristics for which data were extracted (such as study size, PICOS, follow-up period) and provide the citations | Results, Table 1 |
| Risk of bias within studies | 19 | Present data on risk of bias of each study and, if available, any outcome-level assessment (see item 12). | Results |
| Results of individual studies | 20 | For all outcomes considered (benefits or harms), present for each study (a) simple summary data for each intervention group and (b) effect estimates and confidence intervals, ideally with a forest plot |  |
| Synthesis of results | 21 | Present results of each meta-analysis done, including confidence intervals and measures of consistency | Results, Figures 2-6; |
| Risk of bias across studies | 22 | Present results of any assessment of risk of bias across studies (see item 15) | Not applicable |
| Additional analysis | 23 | Give results of additional analyses, if done (such as sensitivity or subgroup analyses, meta-regression) (see item 16) | Not applicable |
| **Discussion** | | | |
| Summary of evidence | 24 | Summarise the main findings including the strength of evidence for each main outcome; consider their relevance to key groups (such as health care providers, users, and policy makers) | Discussion |
| Limitations | 25 | Discuss limitations at study and outcome level (such as risk of bias), and at review level (such as incomplete retrieval of identified research, reporting bias) | Discussion |
| Conclusions | 26 | Provide a general interpretation of the results in the context of other evidence, and implications for future research | Discussion |
| **Funding** | | | |
| Funding | 27 | Describe sources of funding for the systematic review and other support (such as supply of data) and role of funders for the systematic review | Page 18 |

**Appendix 2.** MOOSE checklist

**Biomarkers of inflammation and clinical outcomes in COVID-19 patients: a systematic review and meta-analysis**

| **Criteria** | | **Brief description of how the criteria were handled in the review** |
| --- | --- | --- |
| **Reporting of background** | |  |
| √ | Problem definition | It is uncertain if excess body fat could predict clinical outcomes in COVID-19 patients |
| √ | Hypothesis statement | In patients with COVID-19, do levels of BMI influence clinical outcomes? |
| √ | Description of study outcomes | Mortality, Severe disease, Respiratory failure, Acute respiratory distress syndrome, Poor clinical outcome |
| √ | Type of exposure | BMI |
| √ | Type of study designs used | Observational cohort designs and clinical studies |
| √ | Study population | Adult patients with COVID-19 |
| **Reporting of search strategy should include** | |  |
| √ | Qualifications of searchers | ; Samuel Seidu, MD, Setor K. Kunutsor, PhD |
| √ | Search strategy, including time period included in the synthesis and keywords | Time period: from inception to 16 May 2020  The detailed search strategy can be found in Appendix 3 |
| √ | Databases and registries searched | MEDLINE, Embase, Web of Science and The Cochrane Library |
| √ | Search software used, name and version, including special features | OvidSP was used to search Embase and MEDLINE  Refworks used to manage references |
| √ | Use of hand searching | We searched bibliographies of retrieved papers |
| √ | List of citations located and those excluded, including justifications | Details of the literature search process are outlined in the flow chart. The citation list for excluded studies are available on request. |
| √ | Method of addressing articles published in languages other than English | Not applicable |
| √ | Method of handling abstracts and unpublished studies | Not applicable |
| √ | Description of any contact with authors | None |
| **Reporting of methods should include** | |  |
| √ | Description of relevance or appropriateness of studies assembled for assessing the hypothesis to be tested | Detailed inclusion and exclusion criteria are described in the Methods section. |
| √ | Rationale for the selection and coding of data | Data extracted from each of the studies were relevant to the population characteristics, study design, exposure, and outcome. |
| √ | Assessment of confounding | We assessed confounding by ranking individual studies on the basis of different adjustment levels and performed sub-group analyses to evaluate differences in the overall estimates according to levels of adjustment. |
| √ | Assessment of study quality, including blinding of quality assessors; stratification or regression on possible predictors of study results | Study quality was assessed based on the nine-star Newcastle–Ottawa Scale using pre-defined criteria namely: population representativeness, comparability (adjustment of confounders), ascertainment of outcome. Sensitivity analyses by several quality indicators such as study size, duration of follow-up, and adjustment factors. |
| √ | Assessment of heterogeneity | Results |
| √ | Description of statistical methods in sufficient detail to be replicated | Described in methods section |
| √ | Provision of appropriate tables and graphics | Table 1; Figures 1-5 |
| **Reporting of results should include** | |  |
| √ | Graph summarizing individual study estimates and overall estimate | Figure 2-5 |
| √ | Table giving descriptive information for each study included | Table 1 |
| √ | Results of sensitivity testing | Subgroup analysis showed that good quality studies and advanced age influenced the heterogenity |
| √ | Indication of statistical uncertainty of findings | 95% confidence intervals were presented with all summary estimates |
| **Reporting of discussion should include** | |  |
| √ | Quantitative assessment of bias | The systematic review is limited in scope, as it involves studies with limited information. |
| √ | Justification for exclusion | All studies were excluded based on the pre-defined inclusion criteria in methods section. |
| √ | Assessment of quality of included studies | Brief discussion included in ‘Methods’ section |
| **Reporting of conclusions should include** | |  |
| √ | Consideration of alternative explanations for observed results | Discussion |
| √ | Generalization of the conclusions | Discussed in the context of the results. |
| √ | Guidelines for future research | We recommend large-scale studies when more data becomes available |
| √ | Disclosure of funding source | In “Acknowledgement” section |

**Appendix 3:** MEDLINE literature search strategy

| **** | [# ▲](http://ovidsp.dc1.ovid.com/sp-4.05.0b/ovidweb.cgi?&S=NHCOFPDGJJACBEDKKPBKNGDKDPICAA00&Sort+Sets=descending) | **Searches** | **Results** | **Type** | **Actions** | **Annotations** |
| --- | --- | --- | --- | --- | --- | --- |
|  | | | | | | |
|  | 1 | 2019 novel coronavirus.mp. | 585 | Advanced |  |  |
|  | 2 | 2019-nCoV infection.mp. | 102 | Advanced |  |  |
|  | 3 | 2019-nCoV disease.mp. | 7 | Advanced |  |  |
|  | 4 | COVID-19.mp. | 11815 | Advanced |  |  |
|  | 5 | coronavirus disease-19.mp. | 127 | Advanced |  |  |
|  | 6 | coronavirus disease 2019.mp. | 2011 | Advanced |  |  |
|  | 7 | novel coronavirus.mp. | 2187 | Advanced |  |  |
|  | 8 | SARS-CoV-2.mp. | 3542 | Advanced |  |  |
|  | 9 | COVID-19 virus.mp. | 53 | Advanced |  |  |
|  | 10 | SARS2.mp. | 9 | Advanced |  |  |
|  | 11 | 2019 nCoV.mp. | 690 | Advanced |  |  |
|  | 12 | 2019nCoV.mp. | 3 | Advanced |  |  |
|  | 13 | COVID 19.mp. | 11815 | Advanced |  |  |
|  | 14 | COVID19.mp. | 145 | Advanced |  |  |
|  | 15 | new coronavirus.mp. | 284 | Advanced |  |  |
|  | 16 | SARS CoV-2.mp. | 3542 | Advanced |  |  |
|  | 17 | SARS-CoV.mp. | 6121 | Advanced |  |  |
|  | 18 | 2019-nCoV.mp. | 690 | Advanced |  |  |
|  | 19 | exp Body Mass Index/ | 125222 | Advanced |  |  |
|  | 20 | BMI.mp. | 142474 | Advanced |  |  |
|  | 21 | exp Body Weight/pa [Pathology] | 10213 | Advanced |  |  |
|  | 22 | exp Obesity/co, mo, pa [Complications, Mortality, Pathology] | 58116 | Advanced |  |  |
|  | 23 | exp Death/ or exp "Cause of Death"/ | 191314 | Advanced |  |  |
|  | 24 | exp Patient Discharge/ | 29317 | Advanced |  |  |
|  | 25 | exp Patient Discharge/ | 29317 | Advanced |  |  |
|  | 26 | recover.mp. | 41636 | Advanced |  |  |
|  | 27 | exp Critical Care/ | 57159 | Advanced |  |  |
|  | 28 | exp Ventilation/ or exp Noninvasive Ventilation/ or exp Pulmonary Ventilation/ | 50925 | Advanced |  |  |
|  | 29 | exp Extracorporeal Membrane Oxygenation/ | 10264 | Advanced |  |  |
|  | 30 | ecmo.mp. | 7558 | Advanced |  |  |
|  | 31 | 1 or 2 or 3 or 4 or 5 or 6 or 7 or 8 or 9 or 10 or 11 or 12 or 13 or 14 or 15 or 16 or 17 or 18 | 15735 | Advanced |  |  |
|  | 32 | 19 or 20 or 21 or 22 | 245091 | Advanced |  |  |
|  | 33 | 23 or 24 or 25 or 26 or 27 or 28 or 29 or 30 | 378671 | Advanced |  |  |
|  | 34 | 31 and 32 | 17 | Advanced |  |  |

**Fig. 2** Associations of BMI ≥25 vs. <25 kg/m^2^ with risk of severe illness and mortality in COVID-19 patients

BMI, body mass index; CI, confidence interval (bars); RR, relative risk
